# Basal Cell Carcinoma Treated with High Dose Rate (HDR) Brachytherapy—Early Evaluation of Clinical and Dermoscopic Patterns during Irradiation

**DOI:** 10.3390/cancers13205188

**Published:** 2021-10-16

**Authors:** Tomasz Krzysztofiak, Grażyna Kamińska-Winciorek, Andrzej Tukiendorf, Magdalena Suchorzepka, Piotr Wojcieszek

**Affiliations:** 1 Brachytherapy Department, Maria Sklodowska-Curie National Research Institute of Oncology (MSCNRIO), Gliwice Branch, ul. Wybrzeże Armii Krajowej 15, 44-100 Gliwice, Poland; tomasz.krzysztofiak@io.gliwice.pl (T.K.); piotr.wojcieszek@io.gliwice.pl (P.W.); 2 Skin Cancer and Melanoma Team, Department of Bone Marrow Transplantation and Haematology-Oncology, Maria Sklodowska-Curie National Research Institute of Oncology, Gliwice Branch, ul. Wybrzeże Armii Krajowej 15, 44-100 Gliwice, Poland; 3 Department of Public Health, Wrocław Medical University, ul. Bartla 5, 51-618 Wrocław, Poland; andrzej.tukiendorf@gmail.com; 4 Pathology Department, Maria Sklodowska-Curie National Research Institute of Oncology, Gliwice Branch, ul. Wybrzeże Armii Krajowej 15, 44-100 Gliwice, Poland; m.suchorzepka@gmail.com

**Keywords:** HDR brachytherapy, basal cell carcinoma, dermoscopy

## Abstract

**Simple Summary:**

Basal cell carcinoma (BCC) is the most frequent malignancy of the Caucasian population. High dose rate (HDR) brachytherapy is a re-emerging treatment method for various skin cancers. Dermoscopy is an acknowledged and widely used diagnostic tool providing the bridge between histopathology and clinical examination. Current literature lacks data reporting on the dermoscopic observation of basal cell carcinomas undergoing brachytherapy. In this article, the authors describe clinical and dermoscopic patterns of basal cell carcinomas from 23 patients treated with HDR brachytherapy, and analyse the evolution of BCC structures.

**Abstract:**

Basal cell carcinoma (BCC) is the most frequent malignancy of the Caucasian population. Dermoscopy is an established diagnostic method providing the bridge between clinical and pathological examination. Surface skin high dose rate (HDR) brachytherapy is an organ sparing treatment method used for non-surgical candidates. This prospective study aimed to observe clinical and dermoscopic features and their evolution in 23 patients with pathologically confirmed BCC that have been treated with HDR brachytherapy. In all cases, custom-made surface moulds were used. HDR brachytherapy was performed with 192Ir, dose 45Gy was delivered to the tumour in nine fractions of 5Gy, three times a week. The evolution of clinical and dermoscopic features was followed up at the beginning of treatment, and on the day of every fraction (t1–t9). Dermoscopic evaluation of neoplastic and non-neoplastic structures was based on current diagnostic criteria according to current literature. Univariate logistic regression showed a decreasing number of clinical and pathological features of basal cell carcinoma with every treatment fraction. The effect was more strongly pronounced for cancer-related dermoscopic structures compared with non-neoplastic features. We used multivariate ordinal logistic regression with random effects to prove that the patients’ age corresponds with the tumour’s response to radiation—which may implicate a better response to treatment among older patients. High dose rate brachytherapy decreases the number of clinical and dermoscopic features typical for basal cell carcinoma. The effect is more pronounced among older patients.

## 1. Introduction

Basal cell carcinoma (BCC) is the most frequent skin cancer and most frequent malignancy among Caucasians worldwide [1,2,3]. It is characterized by slow progression and low mortality. Most lesions (80%) are localized on the head region, among which 90% are located on the face [2,4,5]. Diagnostics include anamnesis, clinical and pathological examination. Dermoscopy (epiluminescence microscopy and skin surface microscopy) is an in vivo examination of epidermis and dermis structures. It is an easy to perform, painless, non-invasive, repetitive diagnostic technique allowing doctors to observe neoplastic and non-neoplastic skin lesions in at least 10× magnification in polarized and non-polarized light [6,7]. It gives additional information in the preliminary assessment of tumour morphologic type [8,9,10]. Moreover, this examination helps to determine surgical margins preoperatively [11,12]. It may also be beneficial in ex-vivo examinations for resected lesions [13]. The current literature lacks data concerning dermoscopic feature evaluation of basal cell carcinomas treated with brachytherapy.

Skin surface brachytherapy uses radioactive sources placed close to the tumour, allowing the delivery of high radiation doses to the treatment area with a steep gradient dose in the normal tissues (i.e., conformity). Nowadays, the most commonly used radioactive source in surface brachytherapy is Iridium 192. It delivers a dose over 12Gy per hour, which is defined as a high-dose-rate brachytherapy (HDR BT). One fraction of the treatment usually takes several minutes [14].

In recent years brachytherapy has gained more attention because of its unique features: high conformity—rapid dose drop-off outside of the target area and the possibility of optimizing the radiation dose distribution. Due to the wide range of commercial applicators available or individual applicators (custom made or 3D printed), sub-millimeter precision in dose optimization can be achieved [14].

Surgery remains the primary treatment method of non-melanoma skin cancer (NMSC’s) [15,16]. Mohs surgery is superior to standard excision with the lowest 5 and 10-year recurrence rate [17,18]. Radiotherapy remains an option only for non-surgical candidates, but most guidelines refer only to external beam radiotherapy [15,16]. Other treatment options such as topical therapies (5% imiquimod or 5% fluorouracil) and destructive approaches (curettage, electrocautery, cryotherapy, laser ablation) should be considered only in patients with low-risk superficial BCC. Photodynamic therapy was found to be effective only for superficial BCC and in cases of thin nodular BCC [15,16].

Skin surface HDR BT is recommended for elderly patients, patients with comorbidities and non-surgical candidates. Furthermore, for patients with tumours involving facial aesthetic units, for whom radical surgery would cause massive deformations, may be advised to choose brachytherapy. There is a need for a standardised, non-invasive, fast and easy use method of follow-up, presumably a dermoscopy.

## 2. Materials and Methods

This study aimed to assess dermoscopic features of basal cell carcinoma among patients qualified for brachytherapy, with a subsequent analysis of clinical and dermoscopic patterns of the treated area. Researchers evaluated lesions clinically, obtained macroscopic and dermoscopic photographs of tumours for further comparison. Dermoscopic features occurring, disappearing and changing during exposition to ionizing irradiation were analysed. Acute radiation dermatitis was also monitored and stratified, which allows objective post-treatment observation.

### 2.1. Patients 

Twenty-three T1 and T2 (according to AJCC ed. 8th edition) patients with a median age of 72 years (SD:9.7 years) who received HDR brachytherapy between September 2020 and March 2021 were included in the study. Patients were disqualified from surgery because of age (ranged from 86 to 95 years, 3/23), the poor expected cosmetic outcome of surgery—tumour localization in the central face region (16/23), or due to patient’s choice (4/23). Tumours were localized on the central facial region in 16 cases, lateral face in four cases, scalp in one case and neck in two cases. Details of patients’ clinical, histopathological and therapeutic characteristics are shown in Table 1.

### 2.2. Treatment

The treatment protocol of the Brachytherapy Department of Maria Sklodowska-Curie National Research Institute of Oncology (MSCNRIO), Gliwice Branch, was developed over the last three decades of experience in the field. It has commonalities with The Groupe Européen de Curiethérapie of the European Society for Radiotherapy & Oncology (GEC-ESTRO) recommendations [22], but there are some differences, i.e., maximal surface dose, mould thickness and fractionation. The schedule is nine fractions of 5Gy delivered to the tumour with a 5 mm margin. The treatment was prescribed three times a week to a total dose of 45 Gy. Custom-made surface mould polyacrylamide applicators precisely covering the treatment area were prepared. On the surface of the applicator, plastic tubes were glued for Iridium 192 HDR radioactive source loading. The position of the source and time of treatment was planned by a medical physicist with OncentraBrachy (Elekta AB, Stockholm, Sweden) software (version 4.6.0), using computed tomography images of the treatment area with an attached applicator. The study protocol was approved by the Local Ethics Committee (KB/430-41/20). All patients gave written informed consent.

### 2.3. Clinical Evaluation

Clinical evaluation of basal cell carcinomas was performed according to 12 clinical features at the beginning of the treatment (t1), and before every treatment fraction(t2–t9). Digital photographic images were obtained using DermLite Cam (3Gen,. San Juan Capistrano, CA, USA) digital dermoscopy camera. Presence (1) or absence (0) of 12 clinical features of BCC according to Tognetti et al. classification [23] were described. They are presented in Table 2. 

Moreover, acute radiodermatitis (according to the RTOG/EORTC scale) [21] was observed. Twelve patients experienced grade 0–1 toxicity, two experienced grade 2, two experienced grade 3, and six experienced grade 4 skin radiation toxicity, called acute radiodermatitis.

### 2.4. Histopathologic Assessment

All lesions before brachytherapy were confirmed in histopathological examination. Standard stains with hematoxylin and eosin were used in all cases, and the specimens were assessed by a qualified pathologist (M.S). Due to the presence of heterogeneous tumours, a dominant pattern in the microscopic image was reported according to the WHO classification of tumours (2018) [20].

### 2.5. Dermoscopic Procedure and Image Data Collection

Dermoscopy was performed by a certified medical doctor who is an expert in dermoscopy and an integral member of the skin cancer brachytherapy team. Dermoscopic assessment of skin lesions was performed using the polarized DermLiteFoto dermoscope (3Gen, LLC, San Juan Capistrano, CA, USA) at tenfold magnification. Dermoscopic images (*n* = 603) of 23 BCCs were acquired nine times (t1–t9)—at the beginning of the treatment and on the day of each fraction. Dermoscopic images were captured and saved using the DermLiteCam digital dermoscopy camera with polarized light and then independently analysed by two certified dermoscopists (T.K and G.K-W), who were blinded to any patient/protocol data following the methods of the study. Dermoscopic evaluation of the tumour was performed based on the third consensus of the International Society of Dermoscopy (61 features) [7], and non—neoplastic features according to an expert consensus of the International Dermoscopy Society (overall 31 features) [6] were described as present (1) and absent (0). Moreover, the evaluators were required to score the presence (1) or absence (0) of dermoscopic features including BCC-associated criteria (16) (Table 3) that were selected based current knowledge [7] in modification of Lallas et al. [8] and Tognetti’s et al. classification [23].

Non-neoplastic patterns of the surrounding area of the tumour were described according to the expert consensus of the International Dermoscopy Society [6]. Since the score is not described in Table 3, while analysing the non-neoplastic surroundings of tumour, scale covering tumour was analysed as a non-neoplastic feature. Correlation between clinically observed radiodermatitis and non-neoplastic dermoscopic patterns of area surrounding tumor was analysed.

### 2.6. Statistical Analysis 

The entire photographic databaseoff 21,526 observations including all clinical and dermoscopic structures was analysed in the final statistical assessment. In the statistical analysis we used standard methods. Univariate logistic regression was applied to evaluate the impact of the BT fractions on binary skin diagnostic outcomes. In turn, to estimate the influence of the collected risk factors on the 16 available completed (ranked) number of Lallas/Tognetti’s features and non-neoplastic lesions, a multivariate ordinal logistic model was used. Due to repeated measures with consecutive BT fractions for each patient, the regressions were extended for random effects. The statistical outcomes were expressed by a classical odds ratio (OR) together with a confidence 95% interval (CI 95%) and a *p*-value. The computation was performed in the R platform [24].

## 3. Results

All patients suffered from high-risk basal cell carcinomas (23/23). Twenty tumours were stratified as T1, three as T2 according to AJCC 8th edition [19]. All tumours were observed nine times (t1–t9) before treatment and before every treatment fraction among all patients (the completeness of the Lallas/Tognetti’s features in the consecutive t1–t9 times were: 40%, 41%, 42%, 43%, 40%, 40%, 35%, 32%, and 29%, respectively), while in case of non-neoplastic lesions, analogously: 9%, 9%, 8%, 9%, 9%, 9%, 8%, 8% and 8%.

The list of statistically significant ORs (*p* < 0.05) of the influence of BT fractions on the skin diagnostic outcomes (univariate logistic regression with random effects) is reported in Table 4.

**Figure 1:**
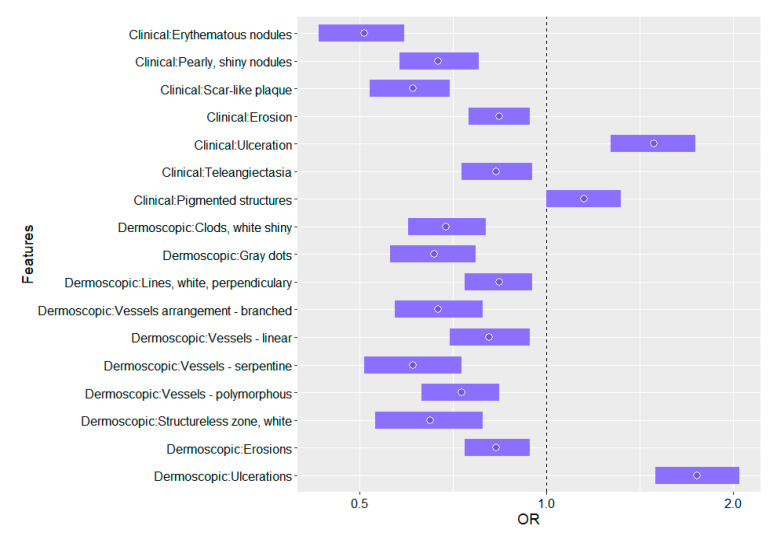
Influence of brachytherapy fractions on skin diagnostic outcomes.

The statistical interpretation of the ORs listed in Table 4 is as follows: each subsequent BT fraction statistically generates a lower chance of occurrence of the erythematous nodules on the skin; the use of one fraction reduces the risk of their occurrence by nearly half, and the use of two doses by (1 − 0.51^2^) × 100% = 74%, i.e., almost three-quarters. In turn, the administered radiotherapy increases the risk of ulceration; the difference of one dose statistically generates an increased risk by almost a half, and for two doses: 1.49^2^ = 2.22, i.e., more than twice. Statistical interpretations of the remaining results reported in Table 4 is analogous. The graphical presentation of ORs of the influence of BT fractions on the skin diagnostic outcomes is shown in Figure 1.

**Figure 2:**
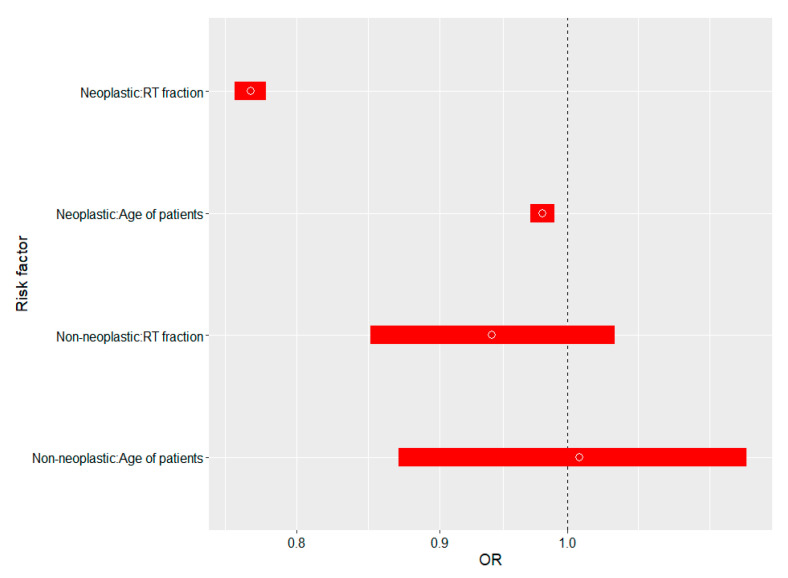
The influence of BT fractions and available risk factors on ranked Tognetti’s clinical (23) and Lallas/Tognetti dermoscopic (8) features of BCCs in correlation with non-neoplastic features (10).

The statistically significant ORs (*p* < 0.05) of the influence of BT fractions and available risk factors on ranked Tognetti’s diagnostic clinical (23) and Lallas/Tognetti’s dermoscopic (8) features (multivariate ordinal logistic regression with random effects) is reported in Table 5 and graphically in Figure 2.

The statistical interpretation of the ORs reported in Table 5 is as follows: each subsequent BT fraction statistically decreases the Lallas/Tognetti’s diagnostic rank, i.e., the first dose of BT by 23%, and two fractions by nearly (1 − 0.77^2^) × 100% ≈ 40. In addition, a 10-year difference in age of patients generates a (1 − 0.98^10^) × 100% = 18% reduction in the cumulative number of clinical and dermoscopic features characterizing tumor, while in case of non-neoplastic features, ORs for the same risk factors were statistically non-significant (*p* > 0.05). The results are shown graphically in Figure 2.

Non-neoplastic dermoscopic patterns of the area surrounding the tumour are presented in Table 6. There is a confirmed negative, statistically significant correlation between intensity (grade) of clinically described acute radiodermatitis (according to RTOG/EORTC criteria) [21] and the number of its non-neoplastic dermoscopic features (OR = 0.65 (0.45,0.93), *p* = 0.017). The number of dermoscopic features decreases with increasing clinical grade of radiodermatitis (*p* = 0.017).

## 4. Discussion

In general, the population nodular subtype of BCC is dominating especially on the head, with 60–80% occurrence. It is followed by a superficial BCC subtype with 10–30% occurrence [25]. Among our group, multiple clinical subtypes of BCC were observed—superficial (5/23), nodular (12/23), scleroderma-like (3/23) and infiltrating (3/23).

The broad spectrum of clinical patterns of basal cell carcinomas translates into multiple histopathological variants. All share common morphological features: basaloid cell aggregates with hyper-coloured cell nuclei and sparse cytoplasm, characteristic stroma and retraction artifact. Recent WHO classification divides basal cell carcinomas into two groups [20]. The first group includes tumours with low recurrence risk: nodular, superficial, pigmented, infundibulocystic with adnexal differentiation and fibroepithelial (Pinkus tumour). The second group contains tumours with high recurrence rate: basosquamous, sclerosing/morpheic, infiltrating, BCC with sarcomatoid differentiation and micronodular. The nodular variant is the most common basal cell carcinoma. It is characterized by large islands of neoplastic cells with peripheral palisading. It is not a homogenous group of tumours. It divides into keratotic nodular BCC—with features of mature keratosis in central parts of neoplastic cell islands, nodulocystic BCC—with retrograde cystic changes along islands, adenoid nodular BCC—forming reticular islands with pseudo adenoidal structures. In the studied material nodular variant was dominant (15 cases), among which in three cases pseudo adenoidal structures characterizing adenoid cystic subtype were found. In four cases low recurrence risk superficial type was present. In this variant in histopathological examination connection between neoplastic cell nests and the epidermal layer is present. Microscopically cancer foci spread into several locations was also observed, which explains this tumour’s other description—superficial multifocal BCC. High recurrence-risk tumours in the studied group were represented by one micronodular and three infiltrating BCCs. Morphologically micronodular BCC is built from islands of neoplastic cells, however islands are considerably smaller (even <0.15 mm) and are more dispersed. They tend to invade skin nerves, subdermal tissues and muscles. Invasive BCC represents narrow strings and nests of neoplastic cells in rich collagen stroma, especially in front of the tumour. Invasive growth patterns often include neuroinvasion. It shares many similarities with scleroderma-like BCC and is often wrongly recognized as such [20].

In searched literature, only one data concerning dermoscopy in monitoring BCC’s treatment effects using high dose ionizing radiation therapy was reported [26]; Moreover, dermoscopy was also used in the monitoring of changes in the course of lentigo maligna radiotherapy [27], or dermoscopic margin delineation in radiotherapy planning for superficial or nodular basal cell carcinoma [28]. Until the present data concerning dermoscopic features of BCC’s undergoing brachytherapy has not been reported. Dermoscopic follow-up helped to monitor the therapeutic response to selected topical therapies including ingenolmebutate in BCC [29], and systemic therapy with vismodegib in BCC [23]. 

**Figure 3:**
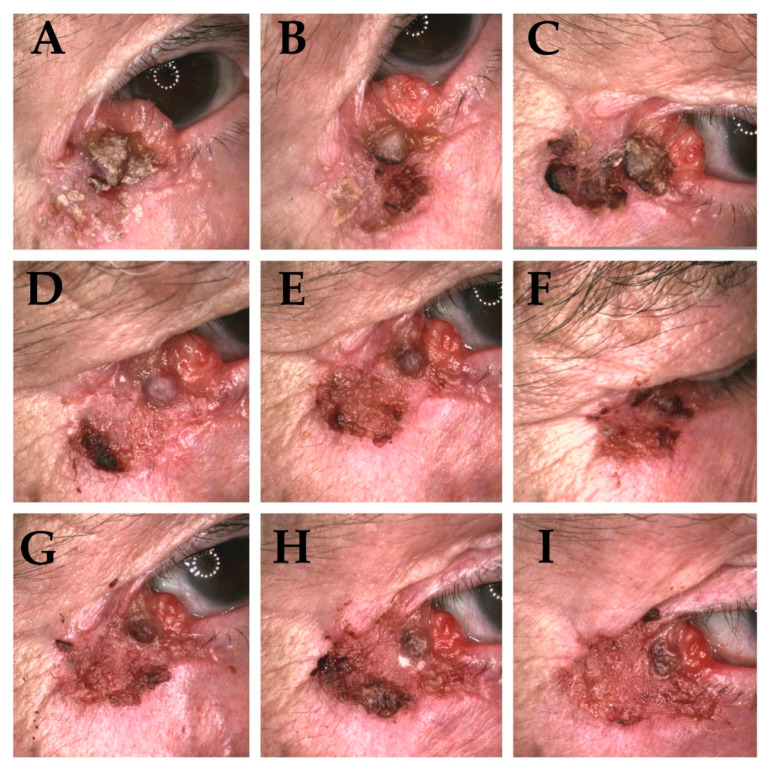
Clinical presentation of basal cell carcinoma of the orbital area of 95-year-old patient, infiltrating type. (**A**)—Tumour prior to brachytherapy, visible—Erythematous nodule, scar-like plaque, pigmented structures, telangiectasia, short vessels, erosion, ulceration. (**B**–**I**) (t2–t9) During subsequent treatment fractions visible gradual vanishing of nodular structures and vessels with formation of ulceration have been noticed.

**Figure 4:**
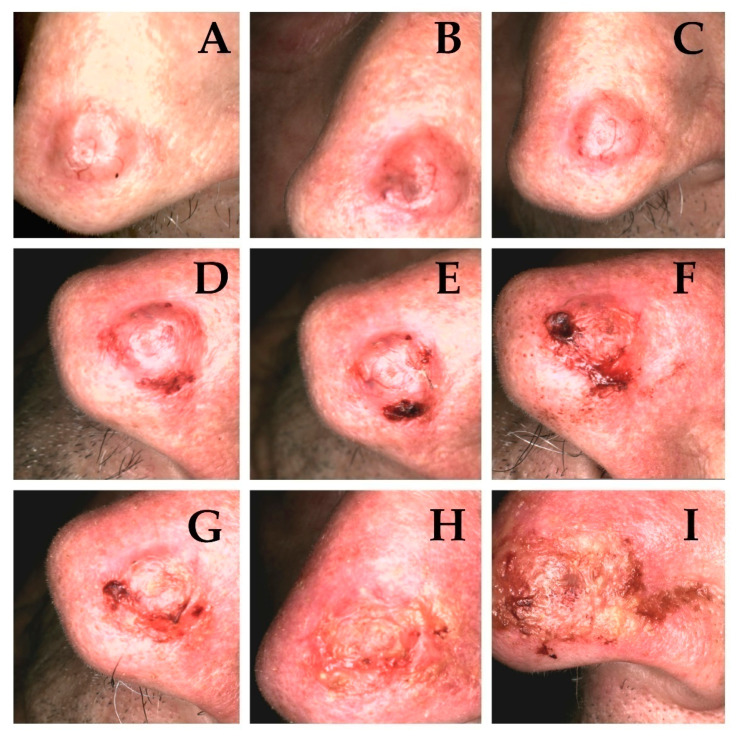
Clinical presentation of basal cell carcinoma of the nose of 83-year-old patient, nodular type. (**A**)—Tumour before treatment, with visible—erythematous nodule, short vessels and telangiectasia. (**B**–**I**)—evolution of tumour destruction with erosion arising in 4th fraction (t4) (**D**), evolving into ulceration with disappearing vessels in 6th fraction (t6) (**F**), and crust appearing in 7th fraction (t7) (**G**), with finally forming ulceration covered by scale and haemorrhagic crusts (t8–9) (**H**,**I**).

Obtained results prove that with every fraction of ionizing radiation the number of clinically visible tumour features diminish. The characteristic clinical appearance of the tumour (erythematous/pearl nodule with e.g., scar-like plaque—depending on the clinical and pathological variant) is replaced by ulceration (Figure 3A–I and Figure 4A–I). 

Destruction of the tumour corresponds with exposure of the underlying stroma, which may explain the increased number of pigmented structures in the irradiated areas. Increased number of pigmented structures indicates exposition of deeper layers of skin after destruction of tumour above. Only further dermoscopic follow up allows to assess the efficacy of the brachytherapy taking into account the persistence of the BCC (total clinical and dermoscopic clearance of the dermoscopic structures of the tumour). Persistence of ovoid nests and bluish dots was observed during monitoring of not efficient treatments reported in the previous publications (i.e., imiquimod applied topically in nodular BBC). Occurrence of these structures during irradiation has an important clinical significance—it raises the need for dermoscopic follow up, but also is an expected symptom of some BCC variants including nodular type, pigmented type previously covered by necrotic tissue or ulceration. Our study exemplifies that dermoscopy is a valuable diagnostic aid that helps to assess potential tumour recurrence/residual disease and to monitor post-brachytherapy response in the future. The presence of pigmented structures such as blue grey ovoid nests as a BCC related features in the previous studies concerning BCC treated with 5% imiquimod suggested poor therapeutic response of BCC [30]. Moreover, the blue-white veil areas and rainbow pattern were only observed in non-responding BCC lesions [31]. The opposite of the above, dermoscopy of responding lesions showed a higher frequency of lesions within focus grey dots [30]. In the final conclusion, the time of the disappearance of blue structures should be taken into account. In the previous study of Husein-ElAhmed [31] blue-grey globules were the fastest to exhibit clearance (50% at week 4), followed by leaf-like areas and large blue-grey ovoid nests [31]. Our short term dermoscopic follow up is a pioneer observation, therefore further investigations are necessary in this matter in the nearest future.

**Figure 5:**
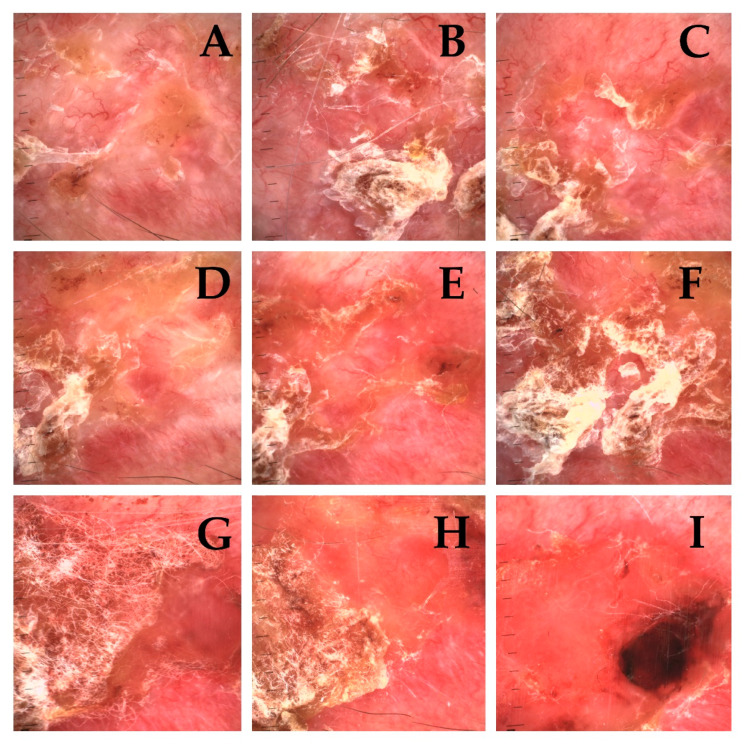
Dermoscopic presentation of basal cell carcinoma, nodular (adenoid type), localized on the forehead of 76-year-old patient. (**A**)—Dermoscopic image before brachytherapy showed the presence of—polymorphous, branched, linear and serpentine vessels, as well as white, shiny clods, perpendicular white lines and white and polychromatic structureless zones, white scale. (**B**–**H**)—Dermoscopy of the BCC during subsequent treatment fractions (t2–t8) revealed diminishing number of serpentine, branched vessels as well as formation of the ulceration arising (t9) (**I**).

**Figure 6:**
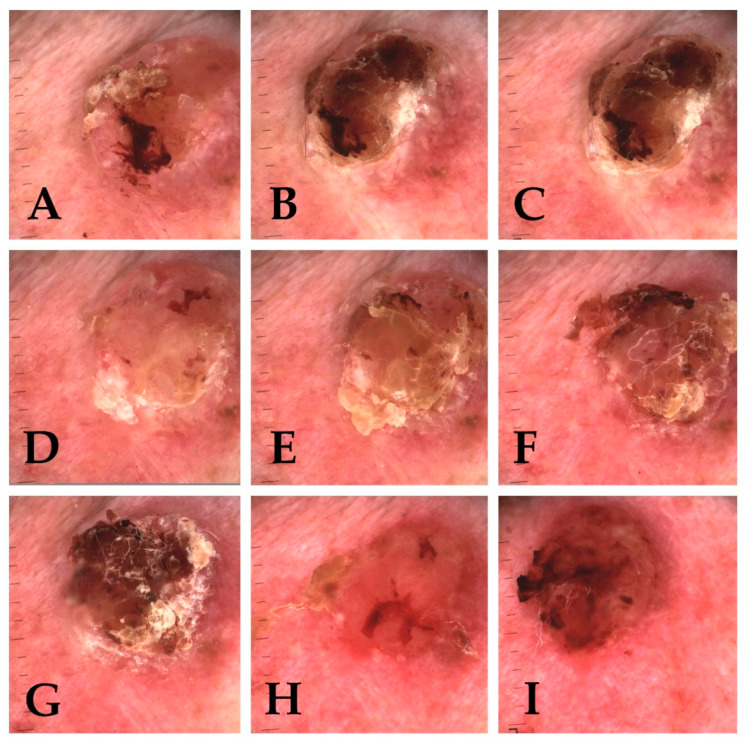
Dermoscopic picture of basal cell carcinoma of the infraorbital region, nodular type, of 67-year-old patient. (**A**)—Tumour prior to brachytherapy (t1) exhibit the presence of white clods, white structureless zones and erosion. (**B**–**I**)—White clods and structureless zones gradually vanishing from 5th fraction (t5), with developing predominance of ulceration.

Dermoscopically higher correlation with the destruction of structures typical for neoplastic tumors (e.g., serpentine vessels) (Figure 5A–I) was observed, than structures also observed in healthy tissue (e.g., linear vessels). According to Reiter’s BCC dermoscopic review, the occurrence of linear vessels in BCC corresponds to all histopathological subtypes of BCC [10]. We found no significant correlation between ongoing brachytherapy and changes in non-neoplastic features, which comparing to strong correlation with changes characteristic for carcinoma brings the conclusion that brachytherapy induces significant effect on the tumour. Clinical and dermoscopic erosion present in tumours often in the beginning of treatment is replaced by ulceration often present in the entire treated area which corresponds with clinical destruction of the treated tumour (Figure 4A–I and Figure 6A–I).

**Figure 7:**
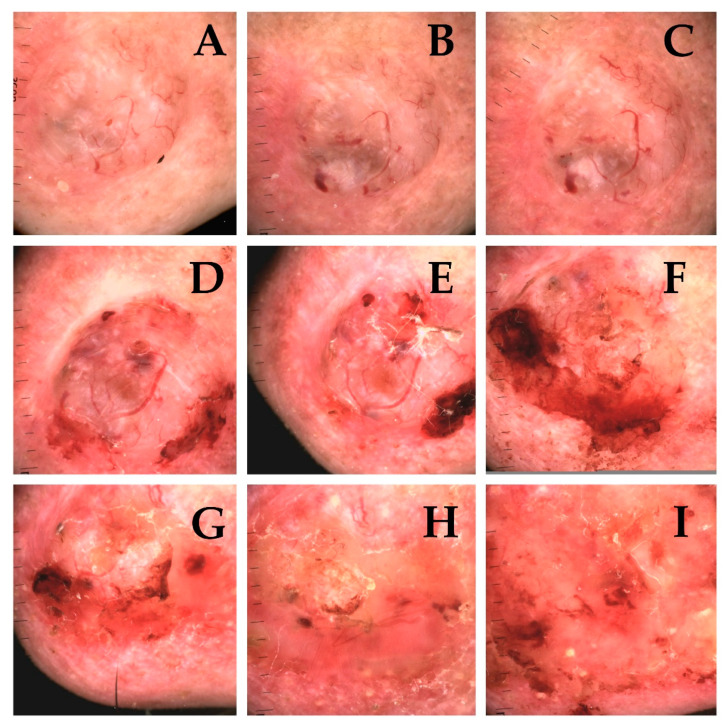
Dermoscopic image of basal cell carcinoma of the nose, nodular type, of 83-year-old patient (clinically presented in Figure 4). (**A**)—tumour prior to brachytherapy (t1), exhibit the presence of multiple white structures including: perpendicular white lines, structureless white zones, white shiny clods, accompanied by multiple polymorphous vessels (serpentine, in branched arrangement), with addition of blue, small clods. (**B**,**C**)—During the consecutive treatment fractions (t2–t3) mentioned structures remain visible. (**D**–**I**) Development of necrosis of the tumour (t4–t9) leads to gradual disappearance of dermoscopic structures with formation of ulceration.

Radiation also reduces the number of structures corresponding with fibrosis including perpendicular white lines, white, shiny clods and white structureless zones (Figure 7A–I). The diminished number of these structures may be the result of image overlapping from ulceration and inflammation.

**Figure 8:**
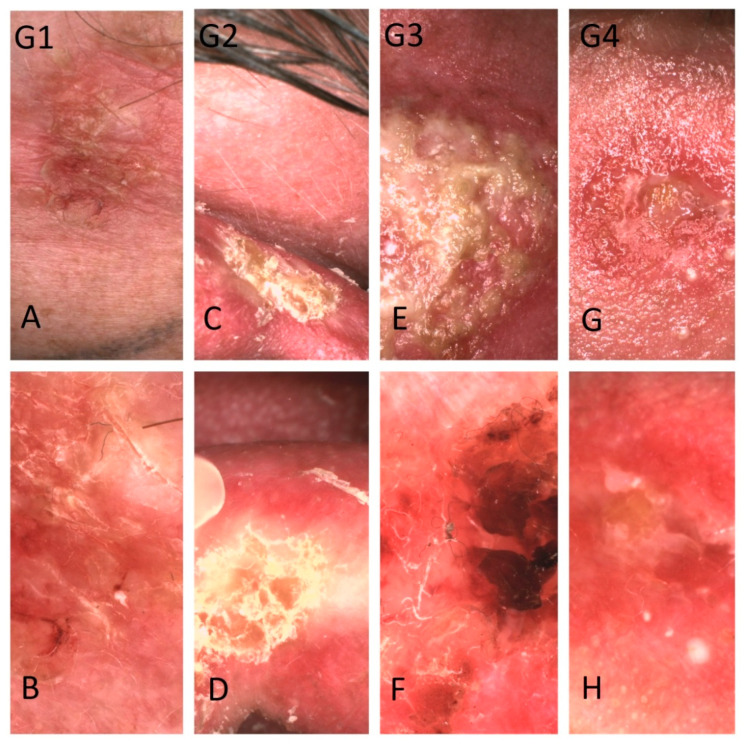
Dermoscopic images of various basal cell carcinomas with coexisting acute radiodermatitis in stage from G1 to G4 were clinically assessed according to RTOG criteria [21] (**A**)—minor erythema of the surrounding area with moderate dry desquamation (G1), (**B**)—dermoscopic image (G1) acute radiodermatitis reveals yellow scale with linear vessels in non-specific distribution in the surrounding area, (**C**)—bright erythema of the tumour’s surroundings with dry desquamation (G2), (**D**)—dermoscopic image of acute radiodermatitis (G2) shows yellow scale (**E**)—confluent, moist desquamation (G3), (**F**)—dermoscopic image (G3) with linear vessels in unspecific distribution, yellow and brown crust. (**G**)—ulceration in acute radiodermatitis (G4), (**H**)—dermoscopic image (G4) reveals the absence of any dermoscopic features.

Observed correlations correspond with the typical radiobiological response of tissue to ionizing radiation [32]. Skin is a hierarchic tissue, and regenerates from a population of stem cells in its deeper layers. Ionizing radiation from a brachytherapy source damages the DNA of stem cells of skin and tumour, without causing much damage to the population of mature cells of upper layers of skin. With time damaged stem cells cannot reproduce a diminishing number of mature cells, leading to ulceration in the irradiated area. Ulceration after tumor destruction heals within three months, from edges into the centre from non-damaged stem cells of healthy skin outside irradiated area [32]. A diminishing number of vessels in the treated area may be caused by radiation damage, but also because ulceration and inflammation may blur the dermoscopic image. Moreover, it is important to distinguish destruction of the tumour from ulceration of healthy skin surrounding BCC. The first effect is desirable, occurs in the second week of treatment. Ulceration of the surrounding healthy skin is an adverse effect defined as an acute radiodermatitis according to RTOG definitions [21]. Exact borders between ulceration created by tumour destruction and radiodermatitis of surrounding skin are often difficult to establish. Dermoscopic non-neoplastic features also correspond with clinically assessed radiodermatitis—i.e., in G2 acute radiodermatitis (according to RTOG/EORTC) diffuse scale is visible among 100% patients. Occurrence of ulceration (G4) corresponds with diminished number of all dermoscopic patterns (Figure 8).

No correlation between non–neoplastic features and brachytherapy in comparison to strong correlation between treatment and changes in BCC specific features indicate that response to radiation is tumour-specific, which also reflects radiobiology—tumouris more susceptible to radiation than healthy tissue [32].

It is worth underlying that all patients in this study suffered from high-risk BCC’s located in the facial ‚H’-zone. According to European expert consensus for management of such lesions [16] primary treatment method should be surgery or radiotherapy for non-surgical candidates. Other destructive therapies including cryosurgery, curettage, electrodessication and laser ablation are dedicated only for small, low-risk non-facial BCC and for multiple small BCCs, whereas photodynamic therapy (PTD) can be considered in non-aggressive, low-risk BCC, i.e., small superficial and nodular types, not exceeding 2 mm tumour thickness, recurrent small and large BCC [16]. Less common histologic variants of BCC, morphoeic, pigmented and micronodular types, as well as areas with higher risk of tumour survival and deep penetration (facial ‘H’-zone), should not be treated with PDT [16].

Correlation between age and higher tissue response to radiation up to now was unexpected. Older patients may be better candidates for brachytherapy than younger since their response to radiation is more pronounced. The correlation between dermoscopic response to radiation and the relapse rate of treated tumours requires further study.

## 5. Limitations of the Study

Dermoscopic evaluation presented in the study did not include post treatment long-term follow up, therefore no data on efficacy of brachytherapy was presented. The observations published above indicate which dermoscopic features diminished during brachytherapy, and which persisted. Further observation is needed to assess clinical significance of those findings and to establish further correlation between the persistence of selected dermoscopic features and the relapse rate of brachytherapy.

## 6. Conclusions

High dose rate brachytherapy decreases the number of clinical and dermoscopic features typical for basal cell carcinoma in the early observation. The effect is more pronounced among older patients.

## Figures and Tables

**Table 1:** Clinical, histopathological and therapeutic characteristics of observed patients’ group.

Sex (M/F)	Median Age	Location of Tumor	Tumour Size According to AJCC [19]	Clinical Subtype [8]	Primary or Recurrent Type	High-Risk and Low-Risk BCC According to NCCN [15]	Histopathological Type (WHO Classification) [20]	Acute Morbidity According to RTOG [21]
10/13	72 (58–95)	Forehead (2) Ear (1) Temporal region (1) Infraorbital region (2) Buccal region (3) Neck (2) Nose (9) Orbital region (2)	T1 (20) T2 (3)	Superficial (5) Nodular (12) Sclerodermiform (3) Infiltrating (3)	Primary (20) Recurrent (3)	High (23)	Nodular (12) Nodular (adenoid) (3) Superficial (4) Micronodular (1) Infiltrating (3)	G0–1 (11) G2 (2) G3 (3) G4 (7)

**Table 2:** Clinical structures of BCC according to Tognetti classification [23].

Clinical Structures	Description	Significance
Pink macules	Well-demarcated pink macules	Superficial BCC
Erythematous papules	Red papules	Nodular BCC
Erythematous nodules	Red nodules	Nodular BCC
Pearly-shiny papules	Translucent papules	Superficial BCC
Pearly-shiny nodules	Translucent nodules	Nodular BCC
Scar-like plaque	Ivory-white plaque resembling scar or morpheaform plaque	Scleroderma-like BCC
Erosions	Multiple small de-epithelized areas	Superficial/nodular BCC
Ulcerations	One or more large red to blackish red areas representing hematogenous crusts	Superficial/nodular BCC
Telangiectasia	Large dilated vessels	Superficial/nodular/scleroderma-like BCC
Short vessels	Short fine vessels	Superficial/nodular/scleroderma-like BCC
Pigmented structures	Hyperpigmented macule, papule, or nodule	Superficial/nodular/scleroderma-like BCC
Crust/scale	Small brown-red to brown-yellow crusts	Superficial/nodular/scleroderma-like BCC

**Table 3:** Dermoscopic structures of BCC-associated criteria (16 features) ranked according to Lallas et al. [8] and Tognetti’s et al. [23] in own modification based on dermoscopic terminology from IDS [7].

Descriptive Terminology	Metaphoric Terminology	Description	Significance
Lines, white, perpendicular	Shiny white streaks (former: chrysalis, chrysalids, crystalline)	Short thick shiny orthogonal crossing lines	Melanoma, BCC, Spitz nevus, dermatofibroma
Lines, radial, connected to a common base	Leaf-like areas	Greyish/bluish brown peripheral globular extensions arising from pigmented network or adjacent confluent pigmented areas	BCC
Lines, radial, converging to a central dot or clod	Spoke wheel area	Brown or greyish well-circumscribed radial projections, usually around a dark brown/black, bluish central axis	BCC
Clods, brown or blue, concentric (clod within a clod)	Concentric globules	Gray/brown/black/blue globular structures with darker central areas	BCC
Clods, blue, large, clustered	Blue-grey ovoid nests	Pigmented ovoid or elongated structures well circumscribed and separate from pigmented tumor body	BCC
Clods, blue, small	Blue globules	Numerous loosely arranged round to oval well circumscribed structures, smaller than nests	BCC
Clods, white, shiny	Shiny white blotches and strands	White structures in the form of circles, oval structures, or large structureless areas, bright white.	BCC
Gray dots	In focus dots	Small well defined loosely arranged grey dots in focus at dermoepidermal junction	BCC
Structureless zone, polychromatic	Rainbow pattern	Many different colours of the rainbow ranging from red to violet	Various diagnoses
Structureless zone, blue	Blue-white veil-like structures	Irregularly margined confluent blue pigmentation with overlying white ground glass haze	Melanoma
Structureless zone, white	Scar-like depigmentation	White structureless areas	BCC presence, tumour fibrotic stroma
Vessel Morphology			
Serpentine	Linear vessels with multiple bends	Linear vessels with multiple bends	Flat BCC, melanoma
Linear	Superficial fine vessels	Telangiectatic vessels in papillary dermis	BCC, inflammation
Vessel Arrangement			
Branched	Arborizing vessels	Bright-red, sharply in focus, large of thick diameter vessels dividing into smaller vessels	BCC
Others	Erosions	Thin crusts overlaying superficial loss of epidermis	BCC
	Ulcerations	Loss of epidermis with/without haematogenous crusts	Various diagnoses

**Table 4:** Statistically significant ORs (*p* < 0.05) of the influence of BT fractions on the skin diagnostic outcomes (univariate logistic regression with random effects).

Clinical Outcome	OR (CI 95%), *p*-Value
Erythematous nodules	0.51 (0.43,0.59), <0.0001
Pearly, shiny nodules	0.67 (0.58,0.78), <0.0001
Scar-like plaque	0.61 (0.52,0.70), <0.0001
Erosion	0.84 (0.75,0.94), 0.0029
Ulceration	1.49 (1.27,1.74), <0.0001
Telangiectasia	0.83 (0.73,0.95), 0.0055
Pigmented structures	1.15 (1.00,1.32), 0.0465
**Dermoscopic Outcome**	**OR (CI 95%), *p*-Value**
Clods, white, shiny	0.69 (0.60,0.80), <0.0001
Gray dots	0.66 (0.56,0.77), <0.0001
Lines, white, perpendicular	0.84 (0.74,0.95), 0.0046
Vessels arrangement—branched	0.67 (0.57,0.79), <0.0001
Vessels—linear	0.81 (0.70,0.94), 0.0049
Vessels—serpentine	0.61 (0.51,0.73), <0.0001
Vessels—polymorphous	0.73 (0.63,0.84), <0.0001
Structureless zone, white	0.65 (0.53,0.79), <0.0001
Erosions	0.83 (0.74,0.94), 0.0028
Ulcerations	1.75 (1.50,2.05), <0.0001

**Table 5:** The influence of BT fractions and available risk factors on ranked Tognetti’s clinical (23) and Lallas/Tognetti’s dermoscopic (8) features of BCCs.

Neoplastic:	Risk Factor	OR (CI 95%), *p*-Value
Yes	BT fraction	0.77 (0.76,0.78), <0.0001
	Age	0.98 (0.97,0.99), 0.0005
No	BT fraction	0.94 (0.85,1.04), 0.2190
	Age	1.01 (0.87,1.16), 0.9440

**Table 6:** Percentage occurrence of dermoscopic non-neoplastic features [6] depending on grade of radiodermatitis according to RTOG/EORTC [21].

TERMINOLOGY		G0	G1	G2	G3	G4
VESSELS	Dotted	0.0%	0.0%	0.0%	0.0%	0.0%
MORPHOLOGY	Linear	24.3%	35.7%	0.0%	50.0%	8.3%
	Branched	24.3%	31.4%	0.0%	50.0%	8.3%
	Curved	4.9%	4.3%	0.0%	0.0%	8.3%
VESSELS	Uniform	2.9%	0.0%	0.0%	0.0%	4.2%
DISTRIBUTION	Clustered	1.9%	0.0%	0.0%	0.0%	0.0%
	Peripheral	1.0%	2.9%	0.0%	0.0%	0.0%
	Reticular	1.9%	7.1%	0.0%	0.0%	4.2%
	Unspecific	19.6%	30.0%	0.0%	50.0%	8.3%
SCALE	White	46.6%	45.7%	25.0%	0.0%	12.5%
COLOUR	Yellow	38.2%	34.3%	100.0%	83.3%	8.3%
	Brown	12.6%	22.9%	50.0%	50.0%	0.0%
SCALES	Diffuse	30.1%	27.1%	100.0%	16.7%	4.2%
DISTRIBUTION	Central	1.9%	11.4%	0.0%	0.0%	0.0%
	Peripheral	20.4%	22.9%	0.0%	0.0%	8.3%
	Patchy	13.6%	11.4%	0.0%	66.7%	0.0%
FOLLICULAR	Plugs	13.6%	15.7%	0.0%	0.0%	8.3%
FINDINGS	Red dots	0.0%	0.0%	0.0%	0.0%	0.0%
	Peripheral White colour	3.9%	2.9%	0.0%	16.7%	0.0%
	Peripheral Pigmentation	5.8%	4.3%	0.0%	0.0%	0.0%
OTHER	White	0.0%	0.0%	0.0%	0.0%	0.0%
STRUCTURES	Brown	0.0%	0.0%	0.0%	0.0%	0.0%
COLOR	Grey	0.0%	0.0%	0.0%	0.0%	0.0%
	Blue	0.0%	0.0%	0.0%	0.0%	0.0%
	Orange	0.0%	0.0%	0.0%	0.0%	0.0%
	Yellow	0.0%	0.0%	0.0%	0.0%	0.0%
	Purple	0.0%	0.0%	0.0%	0.0%	0.0%
OTHER	Structureless	0.0%	0.0%	0.0%	0.0%	0.0%
STRUCTURES	Dots	0.0%	0.0%	0.0%	0.0%	0.0%
MORPHOLOGY	Lines	0.0%	0.0%	0.0%	0.0%	0.0%
	Circles	0.0%	0.0%	0.0%	0.0%	0.0%

## Data Availability

All data used in the study are available from the corresponding author upon request.
